# Objective coagulation assays for thrombotic risk stratification in peripheral artery disease: are we ready for clinical translation?

**DOI:** 10.1097/JS9.0000000000005117

**Published:** 2026-03-17

**Authors:** Aime Ishimwe Mugisha

**Affiliations:** College of Medicine and Health Sciences, School of Medicine and Pharmacy, University of Rwanda, Kigali, Rwanda

*To the Editor,*

Thrombotic complications remain a major clinical challenge in patients with peripheral artery disease (PAD), and objective coagulation assays are emerging as critical tools for precise risk stratification. These assays provide quantitative, reproducible measurements of the coagulation system, capturing the dynamic interactions of platelets, coagulation factors, and fibrinolytic pathways. Unlike conventional tests such as prothrombin time, international normalized ratio, or activated partial thromboplastin time, they offer a more comprehensive assessment of hemostatic function, enabling identification of patients at elevated thrombotic risk^[^1^]^.

The term “objective” reflects the ability of these assays to generate data that are independent of observer interpretation. By integrating multiple components of coagulation, global and factor-specific assays reveal subtle hypercoagulable states that may remain undetected by traditional pathway-specific tests. This quantitative approach supports individualized patient management and informs both clinical and research applications^[^2^]^.

Several platforms exemplify the utility of objective coagulation assays. Thrombin generation assays quantify the total thrombin potential, providing insight into overall coagulability. Viscoelastic testing, including thromboelastography (TEG) and rotational thromboelastometry (ROTEM), evaluates clot kinetics, strength, and lysis in whole blood, offering dynamic information on clot formation and stability. Antifactor Xa activity provides direct monitoring of heparin and related anticoagulants, while platelet function testing assesses responsiveness to antiplatelet therapies. Together, these modalities provide a multidimensional view of thrombotic risk, supporting evidence-based clinical decisions^[^3,4^]^.

The clinical significance of these assays is underscored by recent findings in a cohort of 79 patients with PAD. Among these patients (mean age 71.3 ± 7.1 years, 67.1% men), 20 developed thrombosis. Peak thrombin generation and thrombin generation rate were higher in patients who developed thrombosis. Peak thrombin inversely correlated with citrated kaolin assay reaction time (ρ = −0.33) and citrated kaolin assay + heparinase reaction time (ρ = −0.32), and positively with functional fibrinogen level (ρ = 0.24). Similarly, thrombin generation rate inversely correlated with reaction time (ρ = −0.28), citrated kaolin assay reaction time (ρ = −0.37), and citrated kaolin assay + heparinase reaction time (ρ = −0.39; all *P* < 0.05)^[^1^]^. These observations highlight the ability of objective assays to discriminate patients at higher thrombotic risk, reinforcing their potential role in individualized anticoagulation strategies and precision medicine.

Looking forward, prospective studies are needed to validate assay-driven decision-making in diverse patient populations. Standardization and harmonization of assay protocols across laboratories, as well as incorporation into clinical guidelines, could further enhance their translational impact. By combining precision measurement with clinical insight, objective coagulation assays hold the potential to transform thrombotic risk assessment, improve patient outcomes, and advance vascular medicine^[^5,6^]^. The letter to the editor follows the guidance of the Transparency in the Reporting of Artificial Intelligence in Research (TITAN) guideline^[^7^]^.

## Data Availability

Not applicable. I confirm that the Title Page contains all the required information, and research registration is clearly stated as not applicable.
